# Histopathologic deep learning model for predicting tumor response to hepatic arterial infusion chemotherapy plus TKIs and ICIs in large hepatocellular carcinoma

**DOI:** 10.1186/s40644-025-00885-x

**Published:** 2025-06-06

**Authors:** Chunyu Lin, Yong Ren, Yu Huang, Shuqi Li, Jing Zhang, Shuai Kang, Shurong Li, Changxuan You, Qinghua Cao, Fang Liu

**Affiliations:** 1 State Key Laboratory of Organ Failure Research, Guangdong Provincial Key Laboratory of Viral Hepatitis Research, Department of Infectious Diseases, Department of Liver Tumor Center, Nanfang Hospital, Southern Medical University, No. 1838, Guangzhouda Road, Baiyun District, Guangzhou, 510515 China; 2 Department of Oncology, Nanfang Hospital, Southern Medical University, Guangzhou, Guangdong China; 3 Department of Oncology, Huiqiao Medical Center, Nanfang Hospital, Southern Medical University, No. 1838, Guangzhouda Road, Baiyun District, Guangzhou, Guangdong 510515 China; 4 University of Electronic Science and Technology of China, Chengdu, China; 5 Shensi Lab, Shenzhen Institute for Advanced Study, UESTC, Shenzhen, China; 6 The Seventh Affiliated Hospital of Sun Yat-Sen University, Shenzhen, China; 7 Department of Pathology, The First Affiliated Hospital of Sun Yat-Sen University, Zhongshan Road II #58, Guangzhou, 510080 People’s Republic of China; 8 Department of Radiology, Nanfang Hospital, Southern Medical University, Guangzhou, China; 9 Department of Radiology, The First Affiliated Hospital of Sun Yat-Sen University, Guangzhou, China

**Keywords:** Hepatocellular carcinoma, Hepatic arterial infusion chemotherapy, Tyrosine kinase inhibitors, Immune checkpoint inhibitors, Histopathologic image, Deep learning

## Abstract

**Background:**

While triplet therapy (HTI), which combines hepatic arterial infusion chemotherapy (HAIC) with tyrosine kinase inhibitors and immune checkpoint inhibitors, is widely used in the treatment of large hepatocellular carcinoma (HCC), there are few reports about its efficacy versus HAIC, and no reliable methods are available for promptly predicting HTI response.

**Methods:**

This study included treatment-naïve patients with large HCCs (> 5 cm in diameter) from two centers between January 2017 and December 2022. Objective response rate (ORR), progression-free survival (PFS), and overall survival (OS) were compared between the HTI and HAIC groups. To efficiently predict HTI response, available pre-treatment H&E-stained biopsy slides of HCC patients were collected to develop deep-learning models.

**Results:**

Compared to group HAIC (*n* = 97), group HTI (*n* = 281) showed an ORR (54.45% vs. 21.65%), PFS (median, 10.9 vs. 4.9 months), and OS (median, 25.0 vs. 12.0 months). No significant differences in ORR were observed within the HTI group across different BCLC stages. A deep learning model, termed the Hepatocellular Carcinoma Artificial Intelligence Prediction Model (HAIM), was developed using pathological slides of HTI-treated patients (*n* = 194). HAIM achieved AUC scores of 0.778 (entire testing set), 0.735 (internal testing set), and 0.853 (external testing set).

**Conclusions:**

Integrating TKIs and ICIs with HAIC significantly improved ORR, PFS, and OS in all stages of large HCCs. HAIM, derived from histopathological images of the biopsy, showed potential clinical aid for predicting HTI response, providing a novel tool for personalized management of HCC.

**Supplementary Information:**

The online version contains supplementary material available at 10.1186/s40644-025-00885-x.

## Introduction

Hepatocellular carcinoma (HCC) is the sixth most common cancer worldwide and the second leading cause of mortality in China [1], with nearly 370,000 new cases in 2022 [2], ranking first in the world. Unfortunately, most patients are initially diagnosed with large tumors in developing countries [1].

Hepatic arterial infusion chemotherapy (HAIC) is a promising option for large HCCs that can greatly shrink intrahepatic tumors by delivering high-dose cytotoxic drugs directly to tumors via hepatic artery infusion [3]. It achieves higher conversion rates and fewer adverse events (AEs) than transcatheter arterial chemoembolization [4], dramatically improving clinical outcomes [5]. In addition, triplet therapy (HTI), combining HAIC with tyrosine kinase inhibitors (TKIs) and immune checkpoint inhibitors (ICIs), has been widely administered in patients with large HCCs from Southeast and East Asian countries, especially in China [6]. Although HTI exhibited a high objective response rate (ORR: 51.4%—88.6%) and overall survival (OS: 17.9–30 months) [7–11], a subset of HCC patients who are unresponsive to HTI, especially as neoadjuvant treatment, might miss the opportunity for surgery. Moreover, there are limited comparative studies on the efficacy of HTI versus HAIC alone. Given the significant costs and potential AEs associated with TKIs and ICIs, it is crucial to identify potential responders and non-responders to HTI.

Artificial intelligence models based on computed tomography (CT) or histopathologic images have attracted scholars'attention regarding diagnosing tumors and predicting treatment efficacy. For instance, CT-based models were applied to identify aggressive HCC subtypes, such as macrotrabecular-massive HCC [12] and microvascular invasion [13], and predict the efficacy of TKIs [14]. Whole-slide imaging (WSI) technology digitizes pathological slides and extracts high-resolution morphologic and microenvironmental features through deep learning (DL), which is also applied for diagnosing HCC [15] and predicting the survival of HCC resection [16]. However, no model has been developed to predict tumor response to HTI. Therefore, this study enrolled patients with large HCCs from two institutions and developed DL models for predicting HTI response, aiming to optimize personalized treatment strategies.

## Materials and methods

### Patients and specimens

**Fig. 1:**
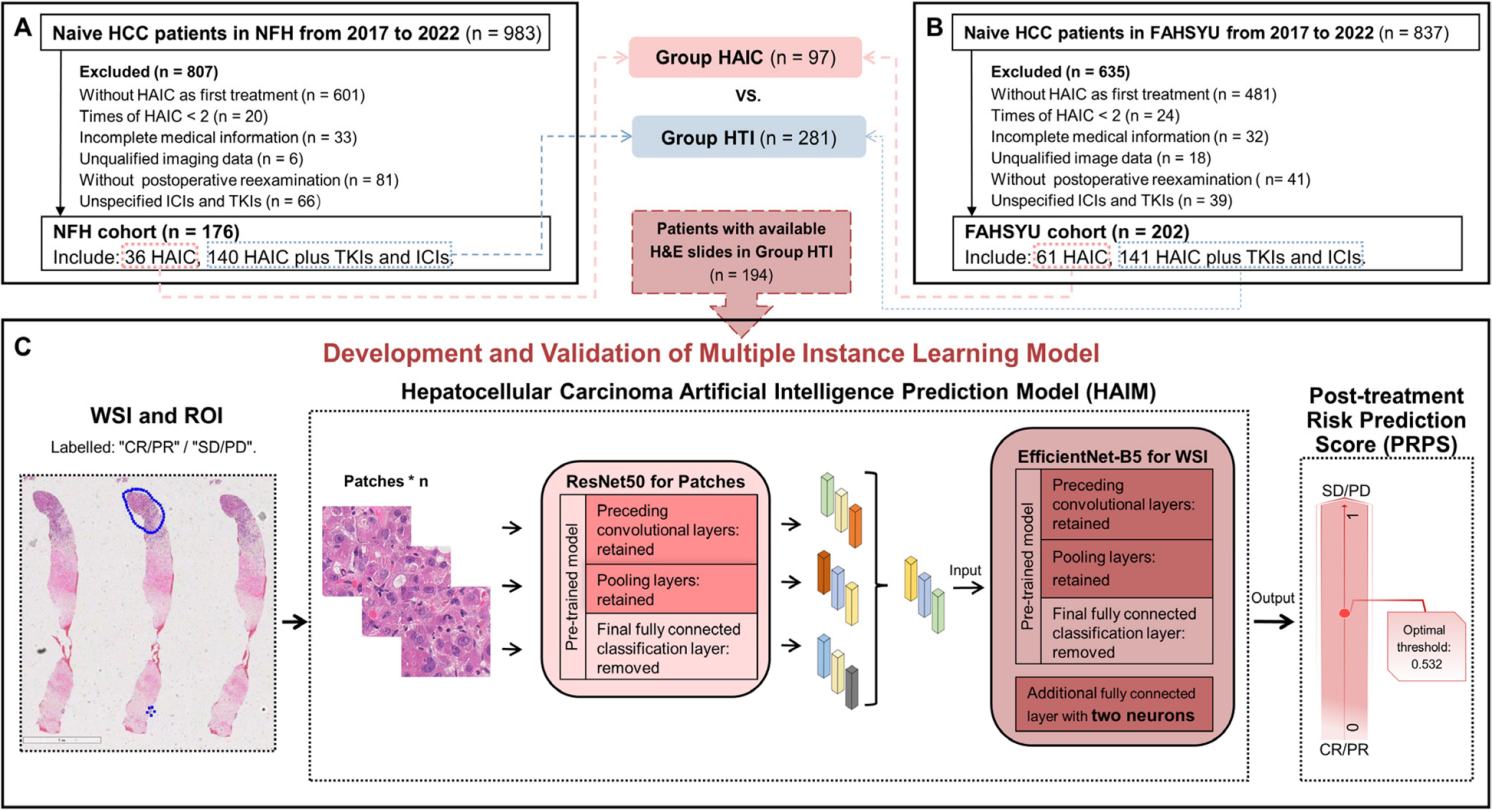
Flow chart. **A** Patient enrollment process for comparing the efficacy and safety of HAIC and HTI in the NFH cohort. **B** Patient enrollment process for comparing the efficacy and safety of HAIC and HTI in the FAHSYU cohort. **C** Overall flowchart of the development of HAIM: Firstly, roughly outline the regions with tumor tissue in the WSI as ROI, and then extract patches within the ROI. Extract features from each patch using the ResNet50 model and aggregate them into a feature vector representing the entire WSI. Utilize multiple-instance learning to model on EfficientNet-B5 to obtain HAIM. Abbreviations: HTI, HAIC in combination with TKIs and ICIs; NFH, Nanfang Hospital; FAHSYU, the First Affiliated Hospital of Sun Yat-sen University; WSI, whole slide imaging; ROI, the regions of interest; CR, complete response; PR, partial response; SD, stable disease; PD, progressive disease

This study enrolled HCC patients at Nanfang Hospital of Southern Medical University (NFH) and the First Affiliated Hospital of Sun Yat-sen University (FAHSYU) between January 2017 and December 2022. Inclusion criteria were defined as follows: (1) diameter of largest intrahepatic lesion > 5 cm; (2) HAIC or HTI as primary treatment and at least 2 cycles of HAIC; (3) availability of baseline contrast-enhanced CT (CECT)/magnetic resonance imaging (MRI); (4) subsequent CECT/MRI evaluation every 2–3 cycles of HAIC post-treatment. Exclusion criteria included: (1) Child–Pugh class C; (2) Eastern Cooperative Oncology Group Performance Scores (ECOG PS) > 2; (3) coexisting other malignant tumors; (4) unidentifiable tumor-feeding artery during HAIC; (5) loss to follow-up or incomplete data (Fig. 1A, B). Baseline clinical characteristics and available pre-treatment hematoxylin and eosin (H&E)-stained biopsy HCC slides were collected.

### HAIC procedure

All HCC patients in this study underwent identical HAIC procedures: under local anesthesia, femoral artery catheterization was performed via the Seldinger technique, followed by hepatic artery angiography to identify the tumor-feeding artery, after which the catheter was positioned super-selectively into the primary tumor-feeding artery and connected to an infusion pump for continuous drug delivery. Oxaliplatin-based HAIC regimen was conducted with cycles repeated every 3 weeks [17].

### ORR on the follow-Up CT and/or MRI, PFS, and OS

Tumor response was classified into four categories: complete response (CR), partial response (PR), stable disease (SD), and progressive disease (PD), according to mRECIST [18]. ORR, disease control rate (DCR), progression-free survival (PFS), OS, and treatment-related AEs were evaluated. Imaging evaluations were independently reviewed by two radiologists blinded to the treatment regimen, with discrepancies resolved by a third expert. AEs were assessed by the US National Cancer Institute Common Terminology Criteria for Adverse Event v5.0 criteria. Follow-up assessments were conducted until disease progression, death, or the study cutoff date (February 29, 2024).

### Preprocessing of histopathologic images and feature representation of patches

All H&E-stained biopsy slides were scanned at 40 × objective magnification using a high-quality whole-slide digital scanner (GScan-20, Guangzhou Light and Shadow Cell Technology Co., Ltd., China). Each WSI underwent quality control to ensure clarity, contrast, and focus were up to standard. Tumor regions were manually outlined as the regions of interest (ROIs) by experienced pathologists using Automated Slide Analysis Platform (v1.9.0). Using the OpenSlide library at 400 × magnification, the ROIs were sampled using a 512 × 512-pixel sliding window, creating a series of non-overlapping patches. Patches that did not contain any tissue cells (background areas) or those that were blurry due to out-of-focus or stained reasons were discarded.

The ResNet-50 architecture was selected as the base model for feature extraction of patches, and parameters that have been fully trained on the ImageNet dataset were directly transferred for rapid model initialization. This pre-trained model's final fully connected classification layer was removed, retaining only the preceding convolutional and pooling layers for feature extraction. For each patch, feature vectors were extracted by passing them through the base model via forward propagation, facilitating subsequent modeling.

### Multiple instance learning with WSI

Each WSI was considered as a "bag" containing n patch instances, with the overall label (“CR/PR” or “SD/PD”) assigned as the bag label. The feature representations of patches were flattened into one-dimensional vectors and concatenated, allowing each bag to be represented as a matrix composed of n rows of instance features. These features were input into a model utilizing EfficientNet-B5 architecture for multiple-instance learning training. Before training, parameters from EfficientNet-B5 that have been fully trained on the ImageNet dataset were directly transferred for rapid initialization. The final fully connected classification layer of this pre-trained model was also removed, retaining only the previous convolutional and pooling layers for feature extraction. An additional fully connected layer with two neurons was appended for CR/PR vs. SD/PD prediction. During training, the Adam optimizer was selected with a learning rate set at 0.01, and the loss function was binary cross entropy. The training process was carried out over 3000 epochs with the best-performing model being saved (Fig. 1C).

### Statistical analysis

IBM SPSS (v26.0), GraphPad Prism (v9.5.0), and R (v4.3.0) were applied for data analysis and statistical visualization. Continuous variables were assessed by Student's t-tests or Mann–Whitney U-tests. Categorical data were evaluated using Fisher's exact or chi-square tests. Variables with *p* < 0.05 in the univariate logistic analysis of ORR were included in multivariate regression. Kaplan–Meier analysis was employed to calculate OS and PFS, with a log-rank test for comparisons. A two-tailed *p*-value of < 0.05 was considered statistically significant.

## Results

### Clinical characteristics and therapeutic efficacy of group HAIC versus HTI

Enrolling 378 patients, a total of 97 patients were allocated to the HAIC group (NFH: 36; FAHSYU: 61) and 281 to the HTI group (NFH: 140; FAHSYU: 141). 51 patients (13.49%) were in BCLC stage A, 75 (19.84%) in stage B, and 252 (66.67%) in stage C. Baseline characteristics, including age, gender, Child–Pugh class, Hepatitis B status, and tumor stage, were balanced between group HAIC and HTI (all *p* > 0.05; Supplementary Table 1, Additional file 1). Median follow-up time was 15.97 months (95% confidence interval [CI]: 14.09–17.84).

**Fig. 2:**
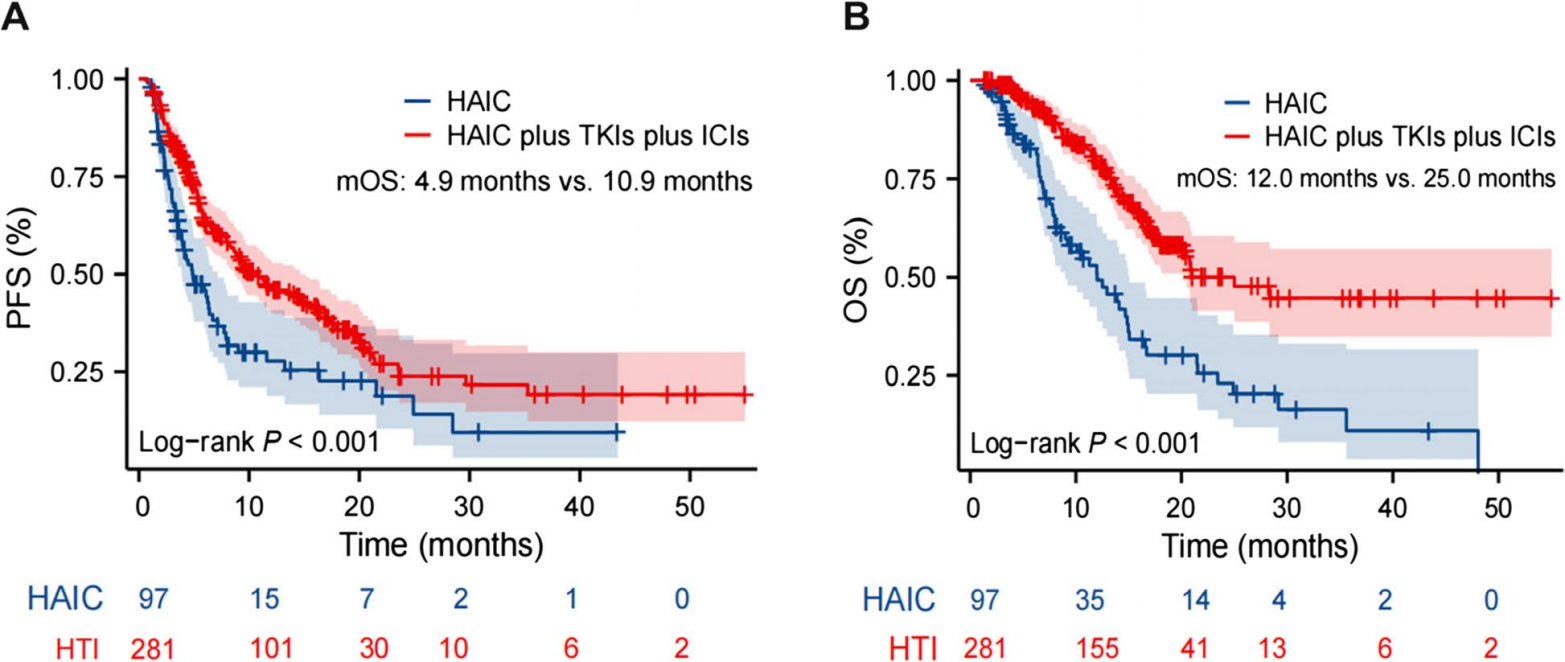
PFS and OS of group HAIC versus HTI in the whole cohort. **A** The HTI group has a significantly longer PFS than the HAIC group (10.9 vs. 4.9 months, *p* < 0.001). **B** HTI group exhibits significantly extended OS than the HAIC group (25.0 vs. 12.0 months, *p* < 0.001)

Group HTI demonstrated significantly higher ORR (54.45% vs. 21.65%, *p* < 0.001) and DCR (93.95% vs. 86.60%, *p* = 0.021) compared to group HAIC (Table 1). ORR did not vary significantly among different BCLC stages in the HTI group (Supplementary Table 2, Additional file 1). At the time of data cutoff, 134 patients (HAIC: *n* = 54; HTI: *n* = 80) had died. Group HTI showed significantly superior PFS than HAIC, with a median PFS of 10.9 months (95% CI: 8.633–14.667) versus 4.9 months (95% CI: 3.900–7.167; HR: 0.572, 95% CI: 0.407–0.802; log-rank *p* < 0.001; Fig. 2A). The median OS of group HTI (25.0 months, 95% CI: 20.167–NA) was also significantly longer than HAIC (12.0 months, 95% CI: 9.000–15.067; HR: 0.382, 95%CI: 0.251–0.580; log-rank *p* < 0.001; Fig. 2B). AEs of any grade were observed in 56.58% (*n* = 159) of HTI and 53.61% (*n* = 52) of HAIC patients, predominantly grades 1–2 (Supplementary Table 3, Additional file 1).

**Table 1:** Tumor response of HAIC group versus HTI group, according to mRECIST

Tumor response	Total (*n* = 378)	HAIC alone (*n* = 97)	HAIC Plus TKIs and ICIs (*n* = 281)	*p*-value
CR	23	5	18	
PR	151	16	135	
SD	175	63	112	
PD	29	13	16	
ORR	46.03%	21.65%	54.45%	** < 0.001**
DCR	92.06%	86.60%	93.95%	**0.021**

*CR* complete response, *PR* partial response, *SD* stable disease, *PD* progressive disease, *ORR* objective response rate, *DCR* disease control rate

### HAIM Model and Post-treatment Risk Prediction Score

To develop a DL model for predicting HTI response, available pre-treatment H&E-stained biopsy slides were collected from 194 HTI-treated HCC patients (NFH: *n* = 103; FAHSYU: *n* = 91). 70% of them were randomly selected as the training set (*n* = 147), and the remaining 30% as the testing set (*n* = 47). For better comparison, the entire testing set was further divided into an internal testing set (NFH subset, *n* = 19) and an external testing set (FAHSYU subset, *n* = 28). There were similar clinical characteristics and tumor burden between training and testing sets (all *p* > 0.05, Table 2), which justified their use for the development and validation of the AI model.

**Table 2:** Basic characteristics of patients in training and testing sets for the prediction models

Characteristics	Training set	Internal testing set	External testing set	*p*-value
Gender, male	131 (89.12)	17 (89.47)	23 (82.14)	0.507
Age, years	52.52 ± 11.74	52.11 ± 12.90	57.18 ± 10.82	0.147
Hepatitis B	130 (88.44)	17 (89.47)	24 (85.71)	0.927
Liver cirrhosis	103 (70.07)	14 (73.68)	18 (64.29)	0.764
Child–Pugh Class				0.394
A	113 (76.87)	12 (63.16)	22 (78.57)	
B	34 (23.13)	7 (36.84)	6 (21.43)	
Tumor size (mm)	107.08 ± 36.43	109.68 ± 44.01	110.64 ± 35.79	0.875
Number of lesions				0.557
single	41 (27.89)	7 (36.84)	10 (35.71)	
multiple	106 (72.11)	12 (63.16)	18 (64.29)	
PVTT	84 (57.14)	7 (36.84)	18 (64.29)	0.158
Extrahepatic metastasis	37 (25.17)	6 (31.58)	7 (25.00)	0.830
BCLC stage				0.680
A	11 (7.48)	3 (15.79)	2 (7.14)	
B	22 (14.97)	3 (15.79)	3 (10.71)	
C	114 (77.55)	13 (68.42)	23 (82.14)	

Values are expressed as mean ± SD and n (%). PVTT, portal vein tumor thrombosis

It was reported that CT-based models predicted the efficacy of HAIC with AUCs [the area under the receiver operating characteristic (ROC) curves] ranging from 0.7 to 0.9 [19, 20]. Regrettably, we failed to construct efficient models for predicting HTI response using CECT images (AUC = 0.626) or clinical features (logistic regression model: AUC = 0.711) (Supplementary Fig. 1, Additional file 1), which might be attributed to the extensive heterogeneity of HCC and complicated drug interactions [21, 22].

**Fig. 3:**
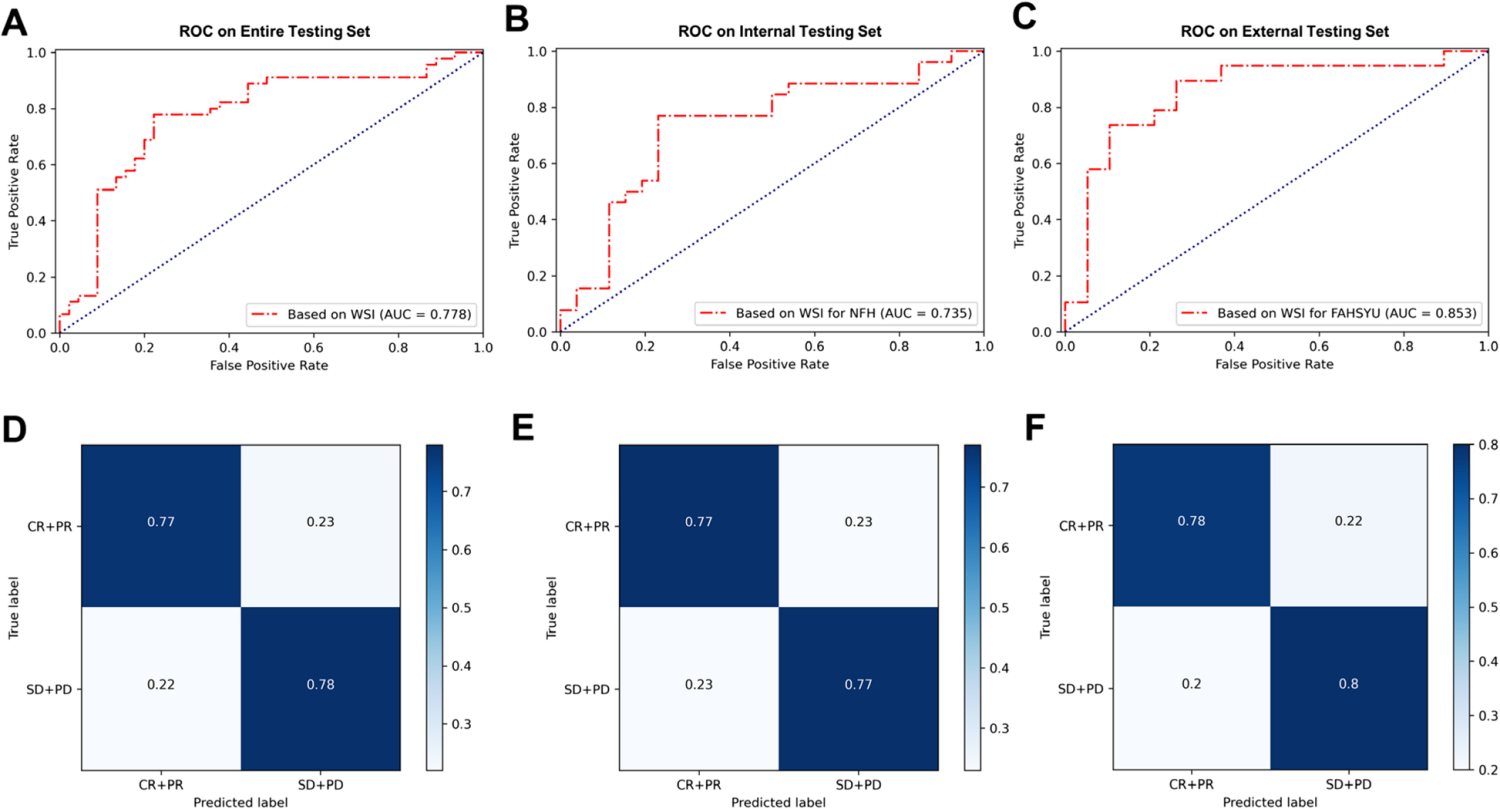
The ROC curves and confusion matrices of the HAIM model. **A**, **B**, **C** HAIM exhibits excellent predictive power, with AUC scores of 0.778, 0.735, and 0.853 for the entire, internal, and external testing sets, respectively. **D**, **E**, **F** The confusion matrices delineate the classification outcomes of HTI response for the entire, internal, and external testing sets, respectively. The darker the blue shading, the more accurate the diagnoses

A histopathologic images-based multiple-instance learning model, termed the Hepatocellular Carcinoma Artificial Intelligence Prediction Model (HAIM), was developed to predict HTI response. HAIM achieved accuracies of 78%, 77%, and 79%, sensitivities of 78%, 77%, and 80%, and AUCs of 0.778, 0.735, and 0.853 in the entire testing set, internal testing set, and external testing set, respectively (Table 3, Fig. 3). The prediction output from HAIM was converted to a Post-treatment Risk Prediction Score (PRPS), ranging from 0 (favoring CR/PR) to 1 (favoring SD/PD). The optimal threshold for this binary classification problem was 0.532, derived from the ROC curves. It represented the point where the difference between the true positive rate and the false positive rate was maximized, aiming to balance sensitivity and specificity.

**Table 3:** Prediction performance of HAIM for different testing sets

Data source	AUC	Accuracy	PPV	NPV	Sensitivity	Specificity	F1 score
Entire testing set	0.778	0.78	0.78	0.77	0.78	0.77	0.78
Internal testing set	0.735	0.77	0.77	0.77	0.77	0.77	0.77
External testing set	0.853	0.79	0.8	0.78	0.80	0.78	0.79

*AUC* the area under the ROC curve, *PPV* positive predictive value, *NPV* negative predictive value

## Discussion

Due to nonspecific symptoms, 70%−80% of HCC patients are diagnosed at an intermediate-advanced stage with large tumors, losing access to curative options and resulting in poor prognosis in China [23]. In our study, HTI improved ORR (54.45% vs. 21.65%), mPFS (10.9 vs. 4.9 months), and mOS (25.0 vs. 12.0 months) compared to HAIC in large HCC, which might be attributed to the synergistic effects of chemotherapeutic drugs, TKIs, and ICIs. Chemotherapeutic drugs induce tumor cell death, release tumor antigens, and increase the infiltration of CD4 + and CD8 + T cells, thereby turning"cold tumors"into"hot tumors” [9, 22]. Besides, TKIs not only suppress angiogenesis and induce vascular normalization, facilitating the delivery of chemotherapeutic drugs [21] but also enhance the efficacy of infiltrating immune cells by reducing tumor hypoxia and acidosis [24].

To eliminate sub-clinical lesions and facilitate tumor downstage, neoadjuvant HAIC is recommended for early HCC patients with high-risk recurrence factors before operation in China (risk factors include: single tumor diameter > 5 cm, multiple tumors, etc.) [25, 26]. In this study, all HCC patients exhibited large tumors (diameter > 5 cm) before treatment, and group HTI achieved a mRECIST-ORR of 54.45%, which was comparable to those of previous studies (51.4% and 54.1%) [7, 8]. Furthermore, there was no significant association between ORR and tumor stage in the HTI group, suggesting that tumor response is more inclined to associate with inherited tumor characteristics. Therefore, it is also advisable for HCC patients with large tumors (> 5 cm in diameter) to undergo neoadjuvant HTI.

Histopathologic images contain rich information of tumor microenvironment [27], which is valuable for predicting tumor response in various types of cancer, such as small-cell lung cancer [28] and gastric cancer [29]. DL can extract deep-level features from histopathological images with less labor intensity than conventional machine learning and effectively overcomes the challenges associated with quantitatively measuring cell morphology [30]. Given the vast pixel count of WSIs, processing the full image is challenging for typical computers. Using tumor regions segmented on histopathological slices as input, DL can dramatically streamline analytical tasks and alleviate the computational load of high-resolution histopathology [30]. Building upon these precedents, this study represented the first instance of using a DL model to extract features from H&E biopsy slides to predict HTI response, providing a novel tool for pre-treatment stratification of HTI responders versus non-responders.

However, this study has several limitations: (1) histopathologic images were from liver tumor biopsy, which did not represent the full spectrum of HCC; (2) the preprocessing of WSIs relied on the subjective annotation of physicians and it is important to develop more automated models to enhance objectivity and reproducibility; (3) prospective multi-center study should be conducted to further validate the generalizability of HAIM.

## Conclusions

Integrating TKIs and ICIs with HAIC significantly improved ORR, PFS, and OS in all stages of large HCCs. HAIM, derived from histopathologic images of the biopsy, showed potential clinical aid for predicting HTI response, providing a novel tool for personalized management of HCC.

## Supplementary Information

Additional file 1.

## Data Availability

The data that support the findings of this study are available from the corresponding author, F.L., upon reasonable request.
