# Supplementary material for: Histopathologic deep learning model for predicting tumor response to hepatic arterial infusion chemotherapy plus TKIs and ICIs in large hepatocellular carcinoma
Source: Cancer Imaging. 2025 Jun 6;25:68. doi: 10.1186/s40644-025-00885-x (PMC12144688; doi:10.1186/s40644-025-00885-x)
Supplement: Supplementary file 1 — Additional file 1. [file 40644_2025_885_MOESM1_ESM.docx]

**Supplementary Table 1. Baseline characteristics of group HAIC versus group HTI.**

| **Variables** | **Total (n = 378)** | HAIC alone **(n=97)** | HAIC Plus TKIs and ICIs (n = 281)  **(n=281)** | **p-value** |
| --- | --- | --- | --- | --- |
| Age, years | 52.29 ± 11.57 | 50.97 ± 11.25 | 52.75 ± 11.66 | 0.192 |
| Gender, male | 339 (89.68) | 86 (88.66) | 253 (90.04) | 0.701 |
| ECOG performance status |  |  |  | 0.402 |
| 0 | 307（81.22） | 76 (78.35) | 231 (82.20) |  |
| 1 | 71（17.78） | 21 (21.65) | 50 (17.79%) |  |
| Child-Pugh Class |  |  |  | 0.200 |
| A | 287 (75.93) | 69 (71.13) | 218 (77.58) |  |
| B | 91 (24.07) | 28 (28.87) | 63 (22.42) |  |
| Hepatitis B | 338 (89.42) | 88 (90.72) | 250 (88.97) | 0.628 |
| Liver cirrhosis | 247 (65.34) | 68 (70.10) | 179 (63.70) | 0.253 |
| Cycles of HAIC | 3.00 (2.00, 4.00) | 3.00 (2.00, 4.00) | 3.00 (2.00, 4.00) | 0.078 |
| Number of lesions |  |  |  | 0.250 |
| single | 119 (31.48) | 26 (26.80) | 93 (33.10) |  |
| multiple | 259 (68.52) | 71 (73.20) | 188 (66.90) |  |
| Largest tumor size, mm | 106.00 (78.25, 135.00) | 103.00 (78.00, 132.00) | 109.00 (79.00, 136.00) | 0.334 |
| Portal vein tumor thrombus | 182 (48.15) | 46 (47.42) | 136 (48.40) | 0.868 |
| Extrahepatic metastasis | 92 (24.34) | 25 (25.77) | 67 (23.84) | 0.703 |
| BCLC stage |  |  |  | 0.129 |
| A | 51 (13.49) | 9 (9.28) | 42 (14.95) |  |
| B | 75 (19.84) | 25 (25.77) | 50 (17.79) |  |
| C | 252 (66.67) | 63 (64.95) | 189 (67.26) |  |
| White blood cell, ×10^9^/L | 6.03 (4.65, 7.80) | 5.89 (5.29, 6.77) | 6.09 (4.39, 7.97) | 0.973 |
| Platelet, ×10^9^/L | 188.00 (132.25, 255.50) | 175.00 (145.00, 218.00) | 191.00 (127.00, 277.00) | 0.461 |
| Alanine transaminase, U/L | 38.00 (25.00, 59.00) | 37.00 (23.00, 54.00) | 39.00 (26.00, 62.00) | 0.055 |
| Aspartate transaminase, U/L | 49.00 (31.00, 74.75) | 49.00 (39.00, 68.00) | 48.00 (28.00, 83.00) | 0.616 |
| Serum albumin, g/L | 37.31 ± 5.22 | 37.61 ± 5.16 | 37.21 ± 5.24 | 0.524 |
| Total bilirubin, μmol/L | 13.90 (10.30, 20.10) | 13.30 (8.90, 19.80) | 14.00 (11.00, 20.30) | 0.100 |
| Serum AFP, ng/mL | 64.15 (11.70, 1612.82) | 65.45 (61.24, 99.76) | 59.90 (9.00, 5059.00) | 0.368 |

Note: Values are expressed as mean ± SD, median (1st Quartile, 3rd Quartile), and n (%).

**Supplementary Table 2. Tumor response of patients with different BCLC stages in the HTI group, according to mRECIST**.

| **Tumor response** | **Total (n =281)** | **BCLC A (n = 42)** | **BCLC B (n = 50)** | **BCLC C (n = 189)** | **p-value** |
| --- | --- | --- | --- | --- | --- |
| CR | 18 | 3 | 4 | 11 |  |
| PR | 135 | 21 | 24 | 90 |  |
| SD | 112 | 17 | 22 | 73 |  |
| PD | 16 | 1 | 0 | 15 |  |
| ORR | 54.45% | 57.14% | 56.00% | 53.44% | 0.883 |
| DCR | 93.95% | 95.24% | 100.00% | 92.06% | 0.084 |

CR, complete response; PR, partial response; SD, stable disease; PD, progressive disease; ORR, objective response rate; DCR, disease control rate.

**Supplementary Table 3. Treatment-related adverse events of group HAIC versus HTI, evaluated by CTCAE v5.0.**

| **Adverse Events, n (%)** | **HAIC alone**  **(n = 97)** | | **HAIC Plus TKIs and ICIs**  **(n = 281)** | | **p-value** | |
| --- | --- | --- | --- | --- | --- | --- |
| **Toxicity Grade** | **Any grade** | **Grade 1/2** | **Any grade** | **Grade 1/2** | **Any grade** | **Grade 1/2** |
| Fatigue | 13 (13.40) | 13 (13.40) | 63 (22.42) | 61 (21.71) | 0.056 | 0.075 |
| Fever | 13 (13.40) | 13 (13.40) | 90 (32.03) | 88 (31.32) | **<0.001** | **<0.001** |
| Abdominal pain | 43 (44.33) | 40 (41.24) | 145 (51.60) | 135 (48.04) | 0.217 | 0.246 |
| Nausea | 25 (25.77) | 23 (23.71) | 82 (29.18) | 78 (27.76) | 0.521 | 0.437 |
| Vomiting | 19 (19.59) | 19 (19.59) | 51 (18.15) | 51 (18.15) | 0.753 | 0.753 |
| Diarrhea | 21 (21.65) | 21 (21.65) | 56 (19.93) | 55 (19.57) | 0.717 | 0.660 |
| Hand-foot syndrome | 3 (3.09) | 3 (3.09) | 66 (23.49) | 54 (19.22) | **<0.001** | **<0.001** |
| Rash | 6 (6.19) | 6 (6.19) | 46 (16.37) | 44 (15.66) | **0.012** | **0.018** |
| Proteinuria | 6 (6.19) | 6 (6.19) | 53 (18.86) | 49 (17.44) | **0.003** | **0.007** |
| Hypertension | 0 (0.00) | 0 (0.00) | 67 (23.84) | 60 (21.35) | **<0.001** | **<0.001** |
| Hyperthyroidism | 3 (3.09) | 3 (3.09) | 19 (6.76) | 19 (6.76) | 0.183 | 0.183 |
| Hypothyroidism | 0 (0.00) | 0 (0.00) | 31 (11.03) | 31 (11.03) | **<0.001** | **<0.001** |
| White blood cell counts decreased | 24 (24.74) | 21 (21.65) | 105 (37.37) | 88 (31.32) | **0.024** | 0.070 |
| Neutrophil count decreased | 29 (29.90) | 26 (26.80) | 85 (30.25) | 71 (25.27) | 0.948 | 0.765 |
| Platelet count decreased | 29 (29.90) | 23 (23.71) | 116 (41.28) | 79 (28.11) | **0.047** | 0.400 |
| ALT increased | 30 (30.93) | 24 (24.74) | 129 (45.91) | 113 (40.21) | **0.010** | **0.006** |
| AST increased | 27 (27.84) | 24 (24.74) | 132 (46.98) | 120 (42.70) | **<0.001** | **0.002** |
| Blood bilirubin increased | 11 (11.34) | 11 (11.34) | 50 (17.79) | 50 (17.79) | 0.136 | 0.136 |
| Hypoalbuminemia | 20 (20.62) | 17 (17.53) | 147 (52.31) | 140 (49.82) | **<0.001** | **<0.001** |

ALT, alanine transaminase; AST, aspartate transaminase.

**
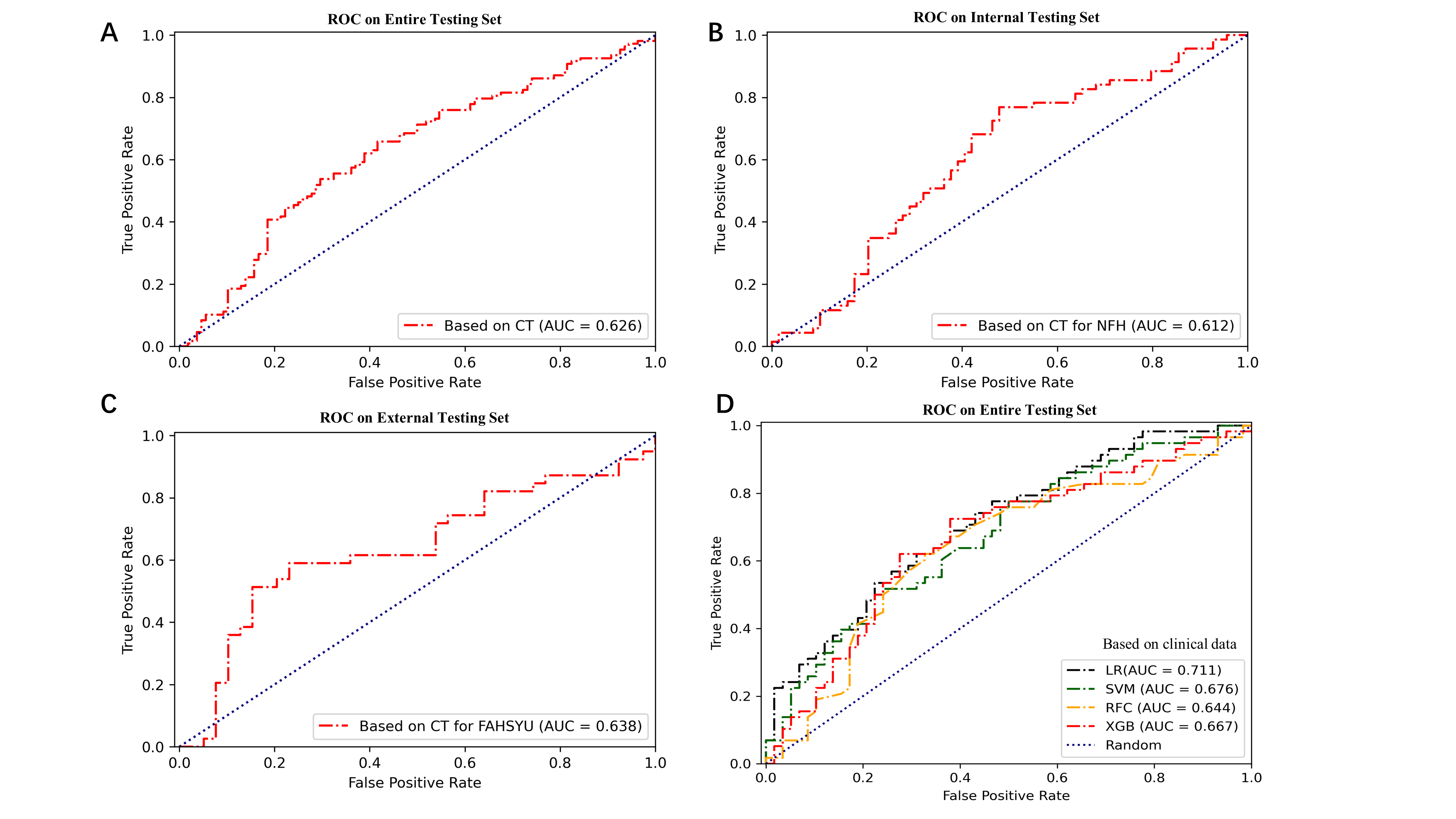
**

**Supplementary Figure 1. The ROC curves for the CT-based model and the clinical data-based model. (A, B, C)** The AUC scores of the CT-based model are 0.626, 0.612, and 0.638 for the entire, internal, and external testing set, respectively. **(D)** The clinical data-based model demonstrates the highest AUC of 0.711 for the entire testing set when employing Logistic Regression (LR), followed by Support Vector Machine (SVM) with an AUC of 0.676, XGBoost (XGB) with an AUC of 0.667, and Random Forest Classifier (RFC) with an AUC of 0.644.
